# Supplementary material for: Pilot study of locomotor asymmetry in horses walking in circles with and without a rider
Source: PeerJ. 2023 Nov 2;11:e16373. doi: 10.7717/peerj.16373 (PMC10625764; doi:10.7717/peerj.16373)
Supplement: Supplemental Information 1 — Hollow side is derived from the questions in Table S2. [file peerj-11-16373-s001.docx]

| Horse | Breed | Gender | Age  (yrs) | Height at  withers (cm) | Duration with  owner (yrs) | Handler  no. | Saddle | Noseband | Bridle | Hollow side |
| --- | --- | --- | --- | --- | --- | --- | --- | --- | --- | --- |
| A | PRE | Stallion | 24 | 155 | 5 | 1 | English | Cavesson | Snaffle | Neither |
| B | Am Curly | Mare | 17 | 153 | 6 | 3 | English | Cavesson | Snaffle | Left |
| C | Russian  crossbred / PRE | Gelding | 17 | 158 | 3 | 1 | English |  | Kimber- wick | Left |
| D | Swedish  warmblood | Gelding | 19 | 167 | 4 | 1 | English | Cavesson | Snaffle | Left |
| F | Lusitano | Gelding | 11 | 150 | 2 | 1 | English | Cavesson | Snaffle | Right |
| H | Swedish  warmblood / PRE | Mare | 14 | 158 | 9 | 3 | Academic | Cavesson | Bitless | Left |
| I | PRE | Stallion | 11 | 153 | 10 | 1 | English | Cavesson | Snaffle | Right |
| J | Friesian | Mare | 9 | 158 | 5 | 6 | Academic | Cavesson | Bitless | Left |
| M | New Forest | Gelding | 6 | 141 | 5 | 5 | Pad | Cavesson | Bitless | Right |
| P | PRE | Gelding | 11 | 155 | 3 | 2 | English | English  noseband | Snaffle | Right |
| Q | PRE | Mare | 8 | 157 | 5 | 1 | English | Cavesson | Snaffle | Neither |
| S | Tinker horse | Gelding | 16 | 152 | 6 | 4 | Academic | Cavesson | Bitless | Right |
| V | Lusitano | Mare | 16 | 154 | 10 | 4 | English | Cavesson | Snaffle | Left |
| X | Iberian cross | Gelding | 8 | 165 | 8 | 1 | English | Cavesson | Snaffle | Right |
| Y | PRE | Gelding | 9 | 158 | 9 | 1 | English | Cavesson | Snaffle | Left |

PRE- Pura raza Espaniol; Handler/rider 6 was the male (and so heavier).
