# Supplementary material for: Pilot study of locomotor asymmetry in horses walking in circles with and without a rider
Source: PeerJ. 2023 Nov 2;11:e16373. doi: 10.7717/peerj.16373 (PMC10625764; doi:10.7717/peerj.16373)
Supplement: Supplemental Information 2 — Questions number 1 and 3 were answered using a modified VAS-scale (1 completely disagree—5 completely agree) and the rest of the questions had the following eligible answer alternatives: left, right (rein for question 2 and direction for other variables), it varies and no-seldom. Column 10 shows the result of the classification and columns 11–15 show whether answers to questions agreed (1) with the resulting hollow designation or not (0). Column 16 shows the occurrences when classification was done from that criterion alone. Note that question number 4 was further stated as ‘the side you achieve a bend in, not the side where you achieve the best bend’. [file peerj-11-16373-s002.docx]

| Horse | 1. I experience that I have equal  contact in the right and left rein when I ride this horse.  1. I completely disagree 2. 3. 4. 5. I completely agree | 2. If your answer is 4 or below,  on which  rein do you usually experience more tension?  Left Right | 3. I think my horse performs   equally well in both directions.  1.I completely disagree. 2. 3. 4. 5. I completely agree | 4. Which side is easier for your  horse to bend to?  Left  Right Equally easy | 5. In which direction does your horse  usually fall into the circle?  Left  Right  It varies  My horse seldomly falls into the circle | 6. In which direction does your horse  usually fall out over the outside shoulder?  Left  Right  It varies  My horse seldomly falls out over the shoulder | 7. In which direction does your  horse usually fall  out with the hind quarters? Left  Right  It varies  My horse seldomly falls out with the hindquarters | Hollow side  (result) | Evaluation 2. Increased rein tension in stiff side rein | Evaluation 4. Easier side to  bend to equals hollow side | Evaluation 5. Falling into circle in stiffer side | Evaluation 6. Falling out over outside shoulder  equals hollow side | Evaluation from  one criterion |
| --- | --- | --- | --- | --- | --- | --- | --- | --- | --- | --- | --- | --- | --- |
| A | 5 |  | 4 | Left | no- seldom | no- seldom | Right | Neither |  | 0 |  |  | 1 |
| B | 3 | Right | 3 | Left | Right | it varies | Left | Left | 1 | 1 | 1 |  |  |
| C | 5 |  | 4 | it varies | no- seldom | Left | no- seldom | Left |  |  |  | 1 | 1 |
| D | 5 |  | 3 | Right | Right | Left | Right | Left |  | 0 | 1 | 1 |  |
| F | 3 | Left | 3 | Right | no- seldom | no- seldom | no- seldom | Right | 1 | 1 |  |  |  |
| H | 5 |  | 3 | it varies | no- seldom | Left | Left | Left |  |  |  | 1 | 1 |
| I | 4 | (Left) | 4 | Right | no- seldom | no- seldom | no- seldom | Right | 1 | 1 |  |  |  |
| J | 4 | Right | 4 | Left | Right | no- seldom | Left | Left | 1 | 1 | 1 |  |  |
| M | 3 |  | 2.5 | Left | Left | Right | Right | Right |  | 0 | 1 | 1 |  |
| P | 2 | (Right) | 2 | Right | Left | Right | Right | Right | 0 | 1 | 1 | 1 |  |
| Q | 5 |  | 5 |  | no- seldom | no- seldom | no- seldom | Neither |  |  |  |  |  |
| S | 4 |  | 3 | Right | Left | Right |  | Right |  | 1 | 1 | 1 |  |
| V | 5 |  | 4 | Equally easy | Right | Left | no- seldom | Left |  |  | 1 | 1 |  |
| X | 2 | Left | 2 | Right | Left | it varies | Left | Right | 1 | 1 | 1 |  |  |
| Y | 2 | Right | 2 | Left | Right | Left | Right | Left | 1 | 1 | 1 | 1 |  |
