# Supplementary material for: Pilot study of locomotor asymmetry in horses walking in circles with and without a rider
Source: PeerJ. 2023 Nov 2;11:e16373. doi: 10.7717/peerj.16373 (PMC10625764; doi:10.7717/peerj.16373)
Supplement: Supplemental Information 3 — Data from 15 horses walking in circles in left (L) and right (R) directions (Dir), including 21 outcome variables as well as speed and circle radius. [file peerj-11-16373-s003.docx]

| Variable | Dir | N | Mean | SD | Min | Median | Max |
| --- | --- | --- | --- | --- | --- | --- | --- |
| Trunk horizontal | L | 1537 | -0.35 | 1.16 | -5.78 | -0.31 | 3.63 |
| (°) | R | 1468 | -0.32 | 1.45 | -5.97 | -0.27 | 4.58 |
| Neck-trunk | L | 1537 | -0.35 | 1.16 | -5.78 | -0.31 | 3.63 |
| (°) | R | 1468 | -0.32 | 1.45 | -5.97 | -0.27 | 4.58 |
| HMaxDiff* | L | 1421 | 6.94 | 26.32 | -69.96 | 4.68 | 111.46 |
| (mm) | R | 1394 | -2.24 | 26.29 | -113.33 | -1.42 | 97.58 |
| WMaxDiff* | L | 1005 | -2.81 | 6.46 | -26.92 | -2.62 | 21.37 |
| (mm) | R | 972 | -0.33 | 6.73 | -20.77 | -0.81 | 29.02 |
| PMaxDiff* | L | 1664 | -2.94 | 5.57 | -21.71 | -3.02 | 20.99 |
| (mm) | R | 1613 | 5.56 | 5.66 | -16.09 | 5.74 | 24.47 |
| HMinDiff* | L | 1420 | -5.95 | 30.09 | -106.3 | -5.26 | 98.66 |
| (mm) | R | 1393 | -10.47 | 30.37 | -113.0 | -8.23 | 83.83 |
| WMinDiff* | L | 1003 | 4.43 | 8.22 | -24.07 | 4.38 | 26.92 |
| (mm) | R | 971 | 0.09 | 8.38 | -22.55 | -0.89 | 24.59 |
| PMinDiff* | L | 1664 | -5.05 | 9.33 | -32.28 | -5.52 | 19.88 |
| (mm) | R | 1613 | 4.55 | 7.95 | -25.46 | 4.57 | 33.94 |
| Pelvic pitch mean | L | 1331 | 81.14 | 12.85 | 57.85 | 81.03 | 106.99 |
| (°) | R | 1258 | 80.89 | 12.99 | 55.65 | 81.03 | 105.59 |
| Pelvic pitch ROM | L | 1327 | 8.23 | 2.21 | 3.57 | 7.95 | 27.83 |
| (°) | R | 1250 | 8.16 | 2.25 | 3.54 | 7.92 | 27.16 |
| Pelvic roll ROM | L | 1425 | 9.40 | 2.55 | 4.17 | 9.10 | 29.24 |
| (°) | R | 1369 | 9.11 | 2.34 | 4.03 | 8.83 | 16.83 |
| Pelvic yaw ROM | L | 1327 | 8.69 | 2.26 | 3.47 | 8.63 | 28.74 |
| (°) | R | 1251 | 8.69 | 2.22 | 3.14 | 8.62 | 25.78 |
| Head ROMz | L | 1582 | 97.32 | 27.18 | 26.24 | 96.56 | 210.3 |
| (mm) | R | 1538 | 95.86 | 27.32 | 28.69 | 94.42 | 188.2 |
| Withers ROMz | L | 1530 | 26.09 | 8.92 | 7.54 | 24.89 | 96.93 |
| (mm) | R | 1461 | 25.95 | 8.23 | 6.71 | 24.85 | 90.23 |
| Pelvic ROMz | L | 1669 | 60.01 | 10.35 | 33.61 | 59.73 | 89.70 |
| (mm) | R | 1613 | 57.72 | 9.51 | 33.86 | 57.57 | 83.46 |
| Hip Inside ROM | L | 1430 | 16.86 | 2.98 | 10.06 | 17.02 | 25.48 |
| (°) | R | 1324 | 18.00 | 2.66 | 10.08 | 17.96 | 25.91 |
| Hip outside ROM | L | 1416 | 20.40 | 3.91 | 10.33 | 20.31 | 31.35 |
| (°) | R | 1356 | 19.20 | 3.63 | 10.21 | 19.43 | 32.35 |
| Stifle inside ROM | L | 1447 | 42.97 | 4.12 | 26.14 | 42.98 | 56.06 |
| (°) | R | 1379 | 41.81 | 3.63 | 28.86 | 41.81 | 51.47 |
| Stifle outside ROM | L | 1352 | 38.69 | 4.27 | 25.19 | 39.16 | 54.94 |
| (°) | R | 1360 | 40.30 | 4.36 | 25.46 | 40.83 | 51.66 |
| Tarsal inside ROM | L | 1462 | 35.99 | 5.31 | 25.19 | 35.59 | 54.05 |
| (°) | R | 1373 | 35.29 | 4.90 | 25.11 | 34.06 | 54.09 |
| Tarsal outside ROM | L | 1348 | 37.27 | 4.40 | 25.23 | 36.64 | 54.45 |
| (°) | R | 1385 | 37.81 | 4.96 | 25.12 | 37.47 | 53.75 |
| Speed | L | 1669 | 1.26 | 0.11 | 0.96 | 1.26 | 1.56 |
| (m/s) | R | 1615 | 1.24 | 0.11 | 0.94 | 1.22 | 1.65 |
| Circle radius | L | 1669 | 4.3 | 0.3 | 3.4 | 4.3 | 4.8 |
| (m) | R | 1615 | 4.3 | 0.4 | 3.5 | 4.3 | 4.9 |

*H (head), W (withers), P (pelvis), maximum / minimum difference in vertical excursion between inside and outside steps.
