# Supplementary material for: Pilot study of locomotor asymmetry in horses walking in circles with and without a rider
Source: PeerJ. 2023 Nov 2;11:e16373. doi: 10.7717/peerj.16373 (PMC10625764; doi:10.7717/peerj.16373)
Supplement: Supplemental Information 4 — Results for all group-level models where speed was significant (p < 0.05). [file peerj-11-16373-s004.docx]

|  | | | Direction models  (Tables 1,2) | | | Models tested  with hollow side  (Tables 3, S5) | | |
| --- | --- | --- | --- | --- | --- | --- | --- | --- |
| Variable | Est | SE | | p | Est | | SE | p |
| Neck-trunk angle (°) | -8.58 | 1.52 | | <0.0001 | -9.65 | | 1.68 | <0.0001 |
| HMinDiff (mm) | -14.2 | 7.25 | | 0.05 |  | |  |  |
| PMinDiff (mm) | 6.80 | 1.88 | | 0.0003 | 7.43 | | 2.06 | 0.0003 |
| Pelvis pitch mean (°) | 1.31 | 0.29 | | <0.0001 | 1.00 | | 0.33 | 0.002 |
| Pelvis roll ROM (°) | 0.42 | 0.04 | | <0.0001 | 0.35 | | 0.05 | <.0001 |
| Pelvis pitch ROM (°) | 0.32 | 0.05 | | <0.0001 | 0.40 | | 0.06 | <0.0001 |
| Pelvic yaw ROM (°) | 0.15 | 0.02 | | <0.0001 | 0.16 | | 0.02 | <0.0001 |
| HROMz (mm) | 4.26 | 0.22 | | <0.0001 | 4.26 | | 0.22 | <0.0001 |
| WROMz (mm) | 0.68 | 0.04 | | <0.0001 | 0.67 | | 0.04 | <0.0001 |
| PROMz (mm) | 0.89 | 0.02 | | <0.0001 | 0.97 | | 0.02 | <0.0001 |
| Hip ROM (°) | 5.15 | 0.36 | | <0.0001 | 5.25 | | 0.40 | <0.0001 |
| Stifle ROM (°) | 212 | 49.5 | | <0.0001 | 262 | | 54.1 | <0.0001 |
| Tarsal ROM (°) | 0.05 | 0.00 | | <0.0001 | 0.05 | | 0.00 | <0.0001 |

HMinDiff - head minimum vertical difference, PMinDiff - pelvis minimum vertical difference, HROMz- head vertical range of motion, WROMz - withers vertical range of motion and PROMz – pelvis vertical range of motion.
