# Supplementary material for: Pilot study of locomotor asymmetry in horses walking in circles with and without a rider
Source: PeerJ. 2023 Nov 2;11:e16373. doi: 10.7717/peerj.16373 (PMC10625764; doi:10.7717/peerj.16373)
Supplement: Supplemental Information 5 — Shown are least square (LS) means, with back-transformation (BTest) where necessary, pairwise ‘between-row’ comparisons and type III p-values. For transformations see Table 2. Data are from seven left and six right-hollow horses, as evaluated from the riders’ answers (Table S2). Coloured cells demonstrate pairwise comparisons ‘between-row’ performed between categories within variables. Black comparisons are significant at p < 0.05 and grey non-significant p ≥ 0.05. [file peerj-11-16373-s005.docx]

| Outcome  variable | Variable categories | | | LS means | | | Between-row | | | | Type III |  |
| --- | --- | --- | --- | --- | --- | --- | --- | --- | --- | --- | --- | --- |
| n | Dir | Limb | Hollo | Est | SE | BTest | comp | | | | Effect | p-value |
| Hip | L | Inside | L | 17.0 | 1.03 |  |  |  |  |  | Speed | <0.0001 |
| ROM | L | Inside | R | 16.7 | 1.11 |  |  |  |  |  | Limb | <0.0001 |
| (°) | L | Outside | L | 21.9 | 1.03 |  |  |  |  |  | Dir | 0.91 |
|  | L | Outside | R | 19.0 | 1.11 |  |  |  |  |  | Dir*limb | 0.0003 |
|  | R | Inside | L | 18.9 | 1.03 |  |  |  |  |  | Hollo | 0.27 |
| 4865 | R | Inside | R | 16.6 | 1.11 |  |  |  |  |  | Limb*hollo | 0.18 |
|  | R | Outside | L | 20.1 | 1.03 |  |  |  |  |  | Dir*hollo | 0.93 |
|  | R | Outside | R | 19.2 | 1.11 |  |  |  |  |  | Dir*limb*hollo | <0.0001 |
| Tarsal | L | Inside | L | 4.90 | 0.01 | 34.0 |  |  |  |  | Speed | <0.0001 |
| ROM | L | Inside | R | 4.92 | 0.01 | 36.8 |  |  |  |  | Condition | <0.0001 |
| (°) | L | Outside | L | 4.92 | 0.01 | 36.6 |  |  |  |  | Dir | 0.87 |
|  | L | Outside | R | 4.92 | 0.01 | 37.4 |  |  |  |  | Limb | <0.0001 |
|  | R | Inside | L | 4.90 | 0.01 | 34.4 |  |  |  |  | Dir*limb | 0.40 |
| 4845 | R | Inside | R | 4.91 | 0.01 | 35.8 |  |  |  |  | Hollo | 0.32 |
|  | R | Outside | L | 4.91 | 0.01 | 35.9 |  |  |  |  | Limb*hollo | 0.49 |
|  | R | Outside | R | 4.93 | 0.01 | 38.6 |  |  |  |  | Dir*hollo | 0.70 |
|  |  |  |  |  |  |  |  |  |  |  | Dir*limb*hollo | 0.003 |

Comp – comparisons, Hollo – hollow.
